# Chitosan/MCM-48 nanocomposite as a potential adsorbent for removing phenol from aqueous solution

**DOI:** 10.1039/d0ra02960b

**Published:** 2020-06-19

**Authors:** Mahmoud Fathy, Hanaa Selim, Abeer E. L. Shahawy

**Affiliations:** Department of Petroleum Application, Core Lab Analysis Center, Egyptian Petroleum Research Institute Cairo Nasr City P.B. 11727 Egypt fathy8753@yahoo.com; Department of Analysis and Evaluation, Central Lab, Egyptian Petroleum Research Institute Cairo Nasr City P.B. 11727 Egypt Hanaaselimali@yahoo.com; Department of Civil Engineering, Faculty of Engineering, Suez Canal University PO Box 41522 Ismailia Egypt abeer_shahawi@eng.suez.edu.eg

## Abstract

A new hybrid mesoporous nanocomposite (CMCM-48) based on chitosan and silica MCM-48 was considered as a potential adsorbent for removing phenol from aqueous solutions (toxic liquid waste) in a batch process. The new composite adsorbent was characterized by scanning electron microscopy (SEM), Fourier transform infrared spectroscopy (FT-IR), X-ray diffraction (XRD), and nitrogen adsorption–desorption isotherms. The adsorption isotherm studies were analyzed using linear and nonlinear Langmuir, Freundlich and Dubinin–Radushkevich models for the optimum conditions when the initial phenol concentration, pH, adsorption temperature and time were 10–500 mg L^−1^, 3–10, 25.5 °C and 300 min, respectively. It was revealed that the experimental results agree well with the Dubinin–Radushkevich model, *i.e.* the correlation coefficient *R*^2^ was 0.983085. The adsorption kinetics was modeled with linear and nonlinear pseudo-first-order, pseudo-second-order and intra particle diffusion kinetic models. The pseudo-second-order model was the best for describing the adsorption process with a correlation coefficient *R*^2^ = 0.99925. The stability of the equilibrium data was studied for a phenol sorbent with a maximum adsorption capacity of 149.25 mg g^−1^. The results verified that the synthesized CMCM-48 was an efficient adsorbent for removing phenol from aqueous solutions.

## Abstract 2

A new hybrid mesoporous nanocomposite (CMCM-48) based on chitosan and silica MCM-48 was considered as a potential adsorbent for removing phenol from aqueous solutions (toxic liquid waste) in a batch process.

## Graphical abstract

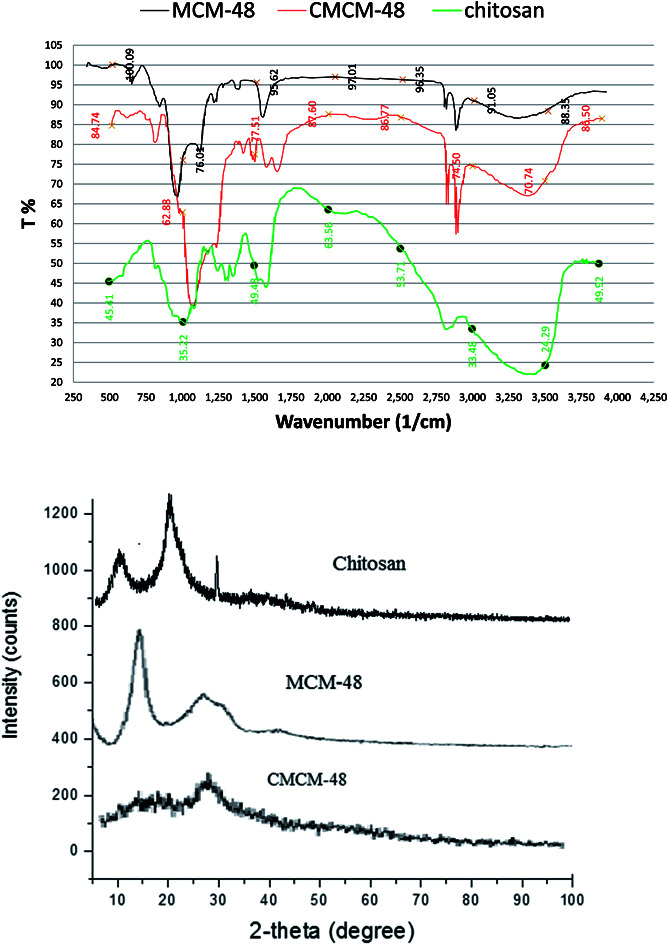

## Introduction

Pollution of drinking water with chemical compounds from different industries is one of the major environmental issues that face humanity. Wastewaters containing organic pollutants such as phenolic compounds represent a serious risk to water quality and human health such as cancer, poisoning, and malformation.^1^ It is commonly known that, among water pollutants, phenolic compounds have poor biodegradability, high toxicity even at low concentrations and high possibility of accumulation within the environment. Accordingly, phenol has been considered as a priority pollutant by the United States Environmental Protection Agency (USEPA) and the National Pollutant material Release Inventory (NPRI) of Canada.^2,3^ Phenol is a weak acid that has a carcinogenic nature and causes environmental problems when it exists in natural water. Phenolic compounds are important for different industrial activities such as oil refining, paint, plastic, petrochemical, pharmaceutical, paper, pulp, and wood products.^4,5^

The discharge of those phenolic compounds into the environment without treatment could result in serious risks to humans, animals, and aquatic systems.^1^ Thus, it is crucial to develop new materials and effective strategies to remove those pollutants from wastewaters. There are several available treatment methods such as electrochemical oxidation, chemical oxidation, extraction, distillation, redox reactions, membrane separation, photocatalytic degradation, precipitation, solvent extraction, and adsorption techniques. Recently, the tendency of phenolic compounds removal involves the adsorption process that could effectively remove a variety of inorganic and organic pollutants from wastewater through three mechanisms, *i.e.*, chemical adsorption, physical adsorption, and ion exchange adsorption.^6^ In the adsorption process, the molecules of the contaminants are preserved on the adsorbent surface and thus may be separated from the water. Various adsorbents have been explored widely for phenol removal such as carbon nanotubes,^7^ activated carbon,^8^ modified diatomite,^9^ chitosan (CS)^10,11^ and mesoporous silica.^12^ Chitosan is one of the most promising adsorbents because chitosan may be considered as a natural compound produced from chitin which has the following advantages: (i) it exists with abundance, (ii) it has brilliant properties such as biocompatibility, biodegradability, and non-toxicity, (iii) its microstructure can be modified in many ways (cross-linking, grafting, functionalization for forming composites, *etc.*). Moreover, chitosan is an efficient sorbent owing to the presence of amino and hydroxyl groups on its polymeric backbone that can serve as chelating and reaction sites between pollutants and chitosan (metals, ions, phenols, dyes, pharmaceutical drugs, pesticides, herbicides, *etc.*).^13^ However, CS can be dissolved in acidic solutions, leading to agglomeration and gel forming, which makes it difficult for CS to disperse and prevents certain hydroxyl and amino groups from chelating metal ions.^14^ To increase the adsorption capacity and enhance the acid resistance, CS is modified by various chemical or physical approaches. Mesostructured inorganic silica as such as CM-48, MCM-41, M, MSU, and SBA are good adsorbents due to their high surface area and pore volume, and uniform pore size.^15^ MCM-48 was considered as one of the famous mesoporous silica materials with an interwoven and branched pore structure which make it an excellent material for adsorbing phenol from aqueous solutions^16^ owing to its regular pore structure, large surface area, and adjustable pore size. The objective of the present work is to explore the possibility of using a new CS/MCM-48 nanocomposite material (CMCM-48) for removing phenol, as organic hazardous waste, from the aqueous wastewater. The new proposed material has the advantage of low-cost and high surface area. The influence of operational parameters such as temperature, pH, contact time and the initial concentration of phenol pollutant solution on the adsorption capacity of CMCM-48was studied. Additionally, the adsorption features were modeled isothermally and kinetically.

## Materials and experimental method

### Chemicals and reagents

Tetraethyl orthosilicate (TEOS, 98%), chitosan, HCl (37%), acetone, acetic acid, glutaraldehyde (Glu), cetyltrimethylammonium bromide (CTAB) and MCM-48 given as (CTS) from Sigma-Aldrich with purity grade 98%.

### Adsorbate and solution

Sigma-Aldrich supplied analytical-reagent grade phenol. The stock solution can be prepared by dissolving, without pH change, the required amount of phenol in dual distilled water. Sequent dilutions were obtained to obtain operating solutions of the desired concentrations.

### Synthesis of mesoporous chitosan–silica composites

The one-pot preparation procedure of TEOS5/5.5–15-CTS2@95 using tetraethyl orthosilicate (TEOS) was used as a silicon precursor. In these notation, the numbers 5/5.5 is the added mass of TEOS in grams, the number 15 is the volume (25%) in ml of Glu, the number 2 is the mass of CTS in grams, and the 95 denotes the aging temperature (°C). The preparation procedures include the following: firstly, TEOS (12.5 g) was dissolved into deionized water (DI, 12.5 g).^17,18^ Secondly, after the reaction mixture was homogenized by stirring for 1 h at 313 K, another portion of the TEOS solution was added dropwise, followed by stirring for 1.5 h. Thirdly, the hydrothermal reaction was statically performed at 368 K (95 °C) for another 20 h. The synthesized nanocomposite was collected by filtration, washed with water/ethanol (40 mL approximately 6 times). Finally, after the addition of chitosan (2.0 g, 0.012 mol) dissolved in acetic acid (50 mL, 2%) and Glu solution (15 mL, 25%), the mixture was stirred for 4.5 h at 313 K (the final sample was dried at 323 K for 10.1 g of the final product, referred to as TEOS 5/5.5–15-CTS2@95).

### Characterization of materials

The CMCM-48 composite was examined using a double beam UV-vis spectrophotometer (Shimadzu, Japan). The pH was estimated by using a pH meter (Eosan). The surface functional groups were detected on a Spectra One FT-IR spectrophotometer in the range 500–4000 cm^−1^. The morphology of the mesoporous materials was examined using a scanning electron microscope (SEM)(JEOL). The concentrations of metal ions coexisted during a mixture solution were determined by inductively coupled plasma-optical emission spectroscopy (ICP-OES) employing a PE-8000 mass spectrometer (PerkinElmer, Wellesley, MA). Elemental analysis of materials was carried out using a Flash EA 1112 elemental analyzer. X-ray diffraction (XRD) was utilized to identify the crystal structure of CMCM-48 samples. XRD patterns were recorded with a Philips 1830 powder X-ray diffractometer, using the Cu-Kα radiation source of wavelength 1.5406 Å for 2 h, ranging from 0.8° to 6.0°, with a 2*θ* step size of 0.01° and a step time of 1 s. Adsorption–desorption isotherms of synthesized samples were obtained at 77 K (−196 °C) on micromeritic model ASAP 2010 sorptometer to determine an average pore diameter. Pore size distributions were calculated by the Barrett–Joyner–Halenda (BJH) method, while the surface area of the sample was measured by the Brunauer–Emmett–Teller (BET) method in the range of relative pressures between 0.05 and 0.20.

### Batch equilibrium isotherm

In adsorption equilibrium, experiments were conducted using the batch contact technique. A set of 250 mL Erlenmeyer flasks, wherever solutions of phenol (200 mL) with varying initial concentrations (10, 50, 250, and 500 mg L^−1^) were added in these flasks. The effect of experimental parameters, such as adsorbent dosage (0.2 mg), pH value (3–10), initial phenol concentration (10–500 mg L^−1^) and contact time (300 min) was studied using batch processing. The influence of equal masses of 0.20 g of CMCM-48 of particle size 150 μm was added to phenol solutions and each sample was kept in an isothermal shaker of 120 rpm at 30 ± 1 °C for 24 h to reach the equilibrium of the solid–solution mixture at constant pH. A similar procedure was followed for another set of Erlenmeyer flask containing the same phenol concentration without CMCM-48 to be used as a blank. Then, the flasks were removed from the shaker. The final phenol concentration in the solution was evaluated using a double beam UV–vis spectrophotometer (Shimadzu, Japan) at *λ*_max_ = 270 nm wavelength. The phenol adsorption capacity *q*_e_ (mg g^−1^) and removal efficiency *R* (%) efficiency were calculated using eqn (1) and (2), respectively:

Adsorption capacity:1
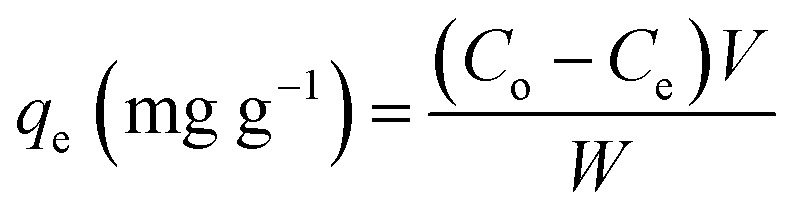


Removal efficiency:^18,19^2
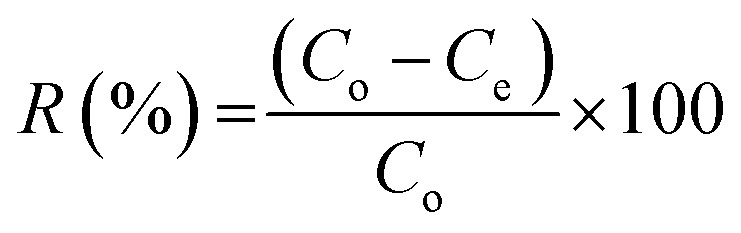
where *C*_0_ is the initial and *C*_e_ is the final concentration in the phenol solutions (mg L^−1^), *V* (L) is the volume of the adsorbate solution, and *W* (g) is the mass of dry adsorbent used. The pH of the solution was adjusted using the required volumes of either 0.1 M HCL and/or 0.1 N NaOH before adding the adsorbent.

The techniques of the kinetic experiments were studied in contrast to those of the equilibrium studies. The aqueous samples were taken at various periods of time, and the phenol concentrations were measured similarly.

## Results and discussion

### Characterization of CMCM-48

**Fig. 1:**
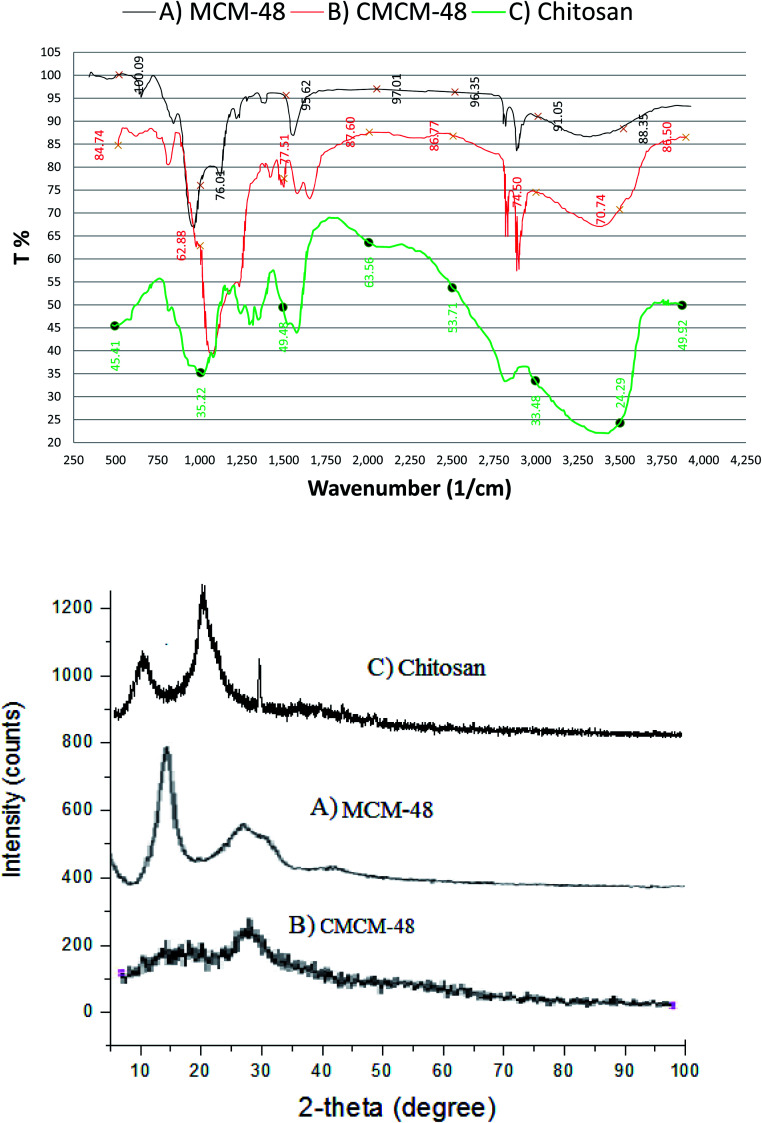
FT-IR spectra and XRD analysis of the synthesized MCM-48 (A), CMCM-48 (B) and (C) chitosan.

The FT-IR spectra of MCM-48 and chitosan were shown in Fig. 1. Fig. 1A reflects the characteristic peaks of MCM-48. The peak at 3400 cm^−1^ is corresponding to O–H stretching vibration in (–COOH group). The peaks at 3005 and 2900 and 1600 cm^−1^ are corresponding to C–H stretching vibration and C=O stretching mode, respectively. The peaks at 1150 and 900 cm^−1^ are associated with Si–O vibration, which corresponds to characteristic peaks of SiO_2_. Fig. 1B shows the FT-IR spectrum of CMCM-48. Besides the SiO_2_, two new peaks at 1600 and 1550 cm^−1^ correspond at the C–O stretching vibration of NHCO and the N–H bending of NH_2_; the large peak at 3440 cm^−1^ was triggered by simultaneous vibrations of O–H and N–H as the –NH_2_ absorption band moved to a lower value.

The absorption of C–O–C stretch appeared at 1152 cm^−1^ and the C–O stretch was found at 1074 cm^−1^. According to the results, the wave number obtained was the characteristic absorption of chitosan. The characteristic spectrum of the chitosan–silica was not different from the spectrum of chitosan as there was only a narrow shift of wave number and addition of new peak. In the spectrum of the chitosan–silica membrane, wave number shifted from 3384 cm^−1^ to 3367 cm^−1^ which indicated the interaction between OH groups of silica and N–H of chitosan. The presence of absorption at 1076 cm^−1^ showed the vibration of Si–O–Si while at 897 cm^−1^, respectively.^20^

FTIR spectra of chitosan are shown in Fig. 1C. The peak observed at 3447 cm^−1^ is attributed to –NH_2_ and –OH groups stretching vibration. This peak is sharper in the chitosan indicating that the hydrogen bonding is enhanced. The peaks at 1657 cm^−1^ and 1598 cm^−1^ are attributed to the CONH_2_ and NH_2_ groups, respectively. The other significant band for CN was observed at 1411 cm^−1^ owing to –CH_2_ wagging.^21^

The XRD pattern of the prepared magnetic MCM-48 is presented in Fig. 1. The result showed relatively well defined XRD pattern for magnetic MCM-48 material, with one strong peak around 2.45 and two small peaks at 4.25 and 4.83 that were assigned to (100), (110), and (200) planes, respectively. Moreover, the fourth peak at 6.44 can be observed and indexed as (210) in the hexagonal system. The observed well-resolved diffraction peaks come from the typical MCM-48.

The XRD pattern of chitosan prepared from waste prawn shells exhibited two characteristic broad diffraction peaks at 2*θ* around 9.63 and 20.53 that are typical fingerprints of semi-crystalline chitosan as indicated in Fig. 1. The peaks around 9.63 and 20.53 are related to crystal-I and crystal-II in chitosan structure and both of these peaks are due to a high degree of crystallinity to the prepared chitosan.^22^

XRD patterns for CMCM-48 are shown in Fig. 1. The obtained characteristic diffraction peaks for CMCM-48 are consistent with those in the literature, which indicated that the mesomorphic orders of MCM-48 remain intact on inclusion of chitosan.^22^ Compared with XRD diffractograms of MCM-41-PAA, however, the apparent decrease in intensity of the (100) diffraction peaks for CMCM-48 in Fig. 1 and other peaks were not observed, suggesting that the ordered structure of nanocomposites was slightly changed due to change of the inherent order caused by the formation of CMCM-48.

**Fig. 2:**
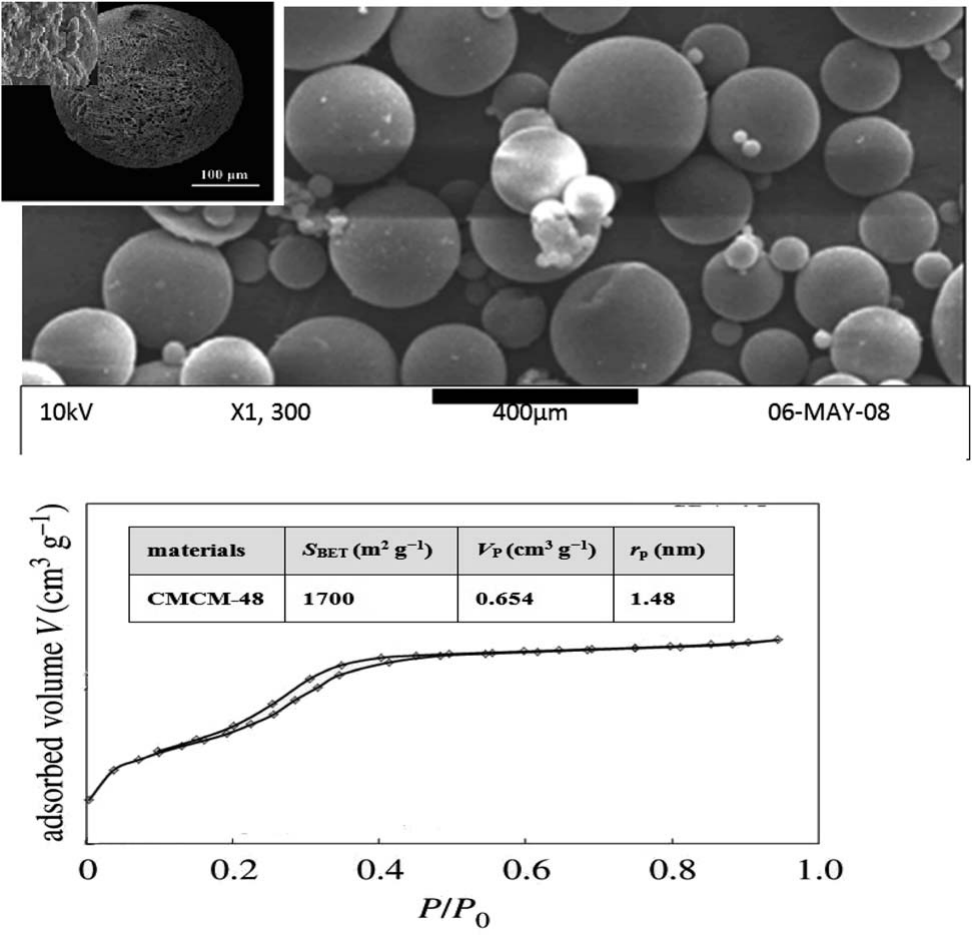
SEM micrograph and N_2_ adsorption–desorption isotherms of CMCM-48 nanocomposite.

As seen in Fig. 2, the SEM image shows the morphology and porosity of the chitosan-based mesoporous silica with a large surface area that could sustain phenol adsorption. The SEM images showed a homogeneous dispersion of the white striped CS with the mesoporous material, forming a spherical structure.

The nitrogen adsorption–desorption isotherms over the surface of the CMCM-48 as well as the pore size distribution curves of various prepared samples are illustrated in Fig. 2. Three well-distinguished regions of the adsorption isotherms are evident: (i) monolayer–multilayer adsorption, (ii) capillary condensation and (iii) multilayer adsorption on the outer particle surface. Apparently, CMCM-48 exhibits type IV isotherm with a distinct capillary condensation step, which is a characteristic pattern of mesoporous materials according to IUPAC classification. N_2_ adsorption–desorption isotherms of CMCM-48 illustrate a clear H1-type hysteresis loop in the relative pressure range between 0.13 and 0.48, implying the presence of very regular mesoporous channels. Collapse of the hysteresis loop is indicated with pronounced narrowing of pore radius, indicating the presence of mesopores.

### Effect of pH value on phenol adsorption

**Fig. 3:**
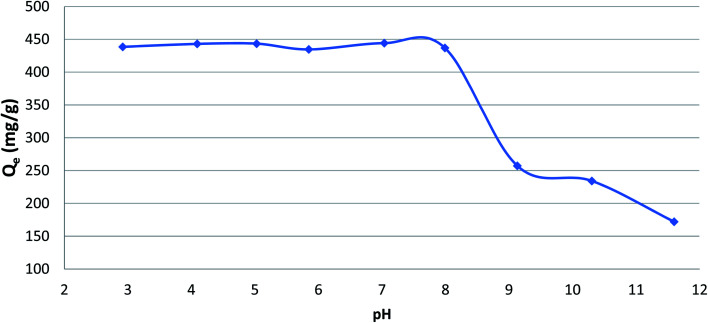
Effect of pH on the adsorption of phenol onto CMCM-48 (initial concentration = 500 mg L^−1^, contact time = 120 min, the adsorbent dosage = 0.1 g, temperature = 25.5 °C).

The initial pH of adsorption phenol played a significant role in the adsorption of phenol onto CMCM-48. To study the influence of pH, all the experiments were performed at a temperature of 25.5 °C and phenol initial concentration of 500 mg L^−1^ using different pH values in the range 3–10. The pH was analyzed by adding few drops of diluted either 0.1 N NaOH or 0.1 N HCl. Fig. 3 illustrates the variation of adsorption capacity with different values of pH, which reveal that the phenol adsorption is maximum up pH value of 8 after which it decreases as the pH increases. This could be attributed to the effect of pH on the surface properties and the degree of ionization of the adsorbate. Here, phenol being as a weak acid with p*K*_a_ value ≈ 9.89 is dissociated at pH > p*K*_a_. The reason could be also due to the electrostatic repulsion force between the negative surface charge of the phenol and the cationic surface of the nanocomposite. While at acidic pH, the percentage removal was higher because phenol was unionized and the dispersion interaction was predominant.

### Effect of temperature

**Fig. 4:**
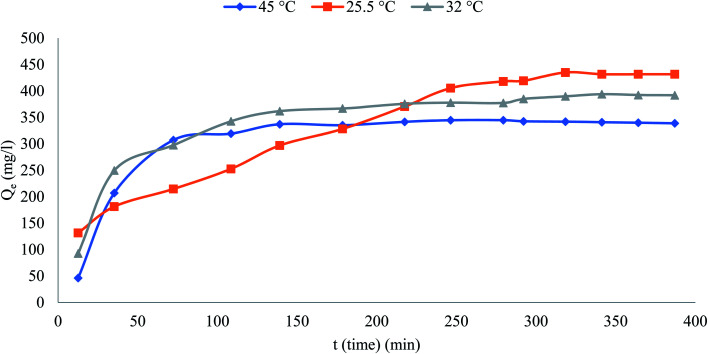
Effect of temperature on removal on phenol% (initial concentration = 500 mg L^−1^, the adsorbent dosage = 0.1 g).

Fig. 4 reveals the influence of temperature on the phenol percentage removal (*R*%)at temperatures 298 and 318 K over the adsorption system from 298 to 318 K to 50 °C.The behavior of the graph shows that the maximum removal was 86.33, 78.38 and 77.77% at temperatures 298, 305 and 318 K respectively. It appears that the decrease in removal percentage of phenol with increasing temperature because of the occurred damage to the active binding sites and weakening of the binding forces between the phenolic and adsorbent molecules.

### Effect of contact time

**Fig. 5:**
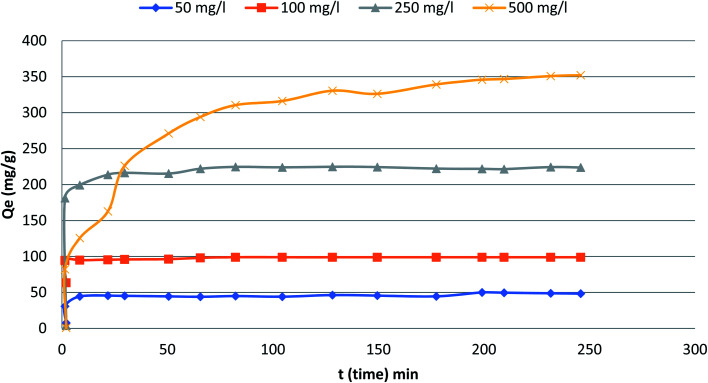
The variation of adsorption with time at different initial phenol concentrations (the adsorbent dosage = 0.1 g, temperature = 25.5 °C).

Fig. 5 depicts the influence of adsorption time in the range from 0 to300 min on the adsorption capacity and removal efficiency of the phenol at various initial concentrations (50, 100, 250 and 500 mg L^−1^). The figure indicates that the adsorption capacity reaches a plateau immediately after a very short contact time especially for initial concentration 50–250 mg L^−1^. For the highest initial concentration, it took slightly longer time to reach the plateau. Eventually, a plateau is attained in all curves indicating that the adsorbent is saturated at this level. The adsorption capacity of phenol increased quickly with increased adsorption time at the initial period. Specifically, under the conditions of initial phenol concentration 500 mg L^−1^ during the initial 80 min and reached equilibrium at around 200 min. The adsorption rates responded quickly due to the accessibility of more adsorption/vacant sites at the initial stage.^23^

### Effect of adsorbent mass

**Fig. 6:**
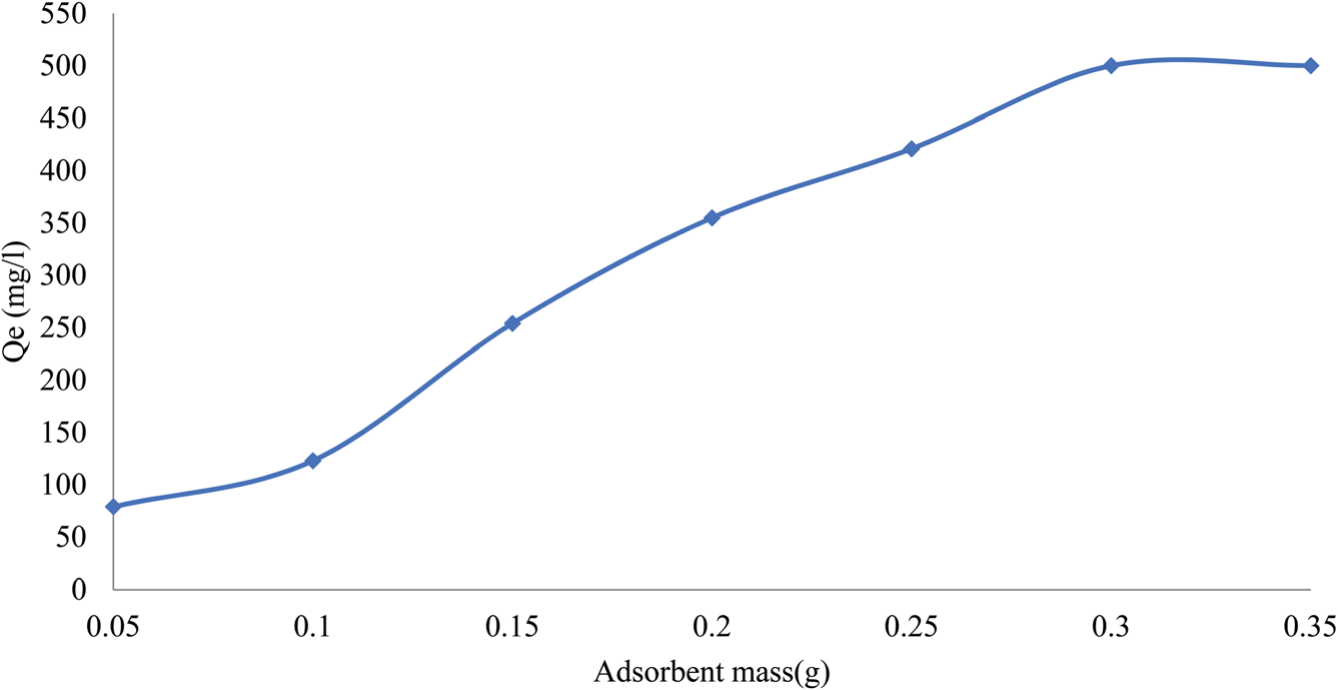
Effect of adsorbent dose on removal efficiency of pollutants by CMCM-48 (contact time = 120 min, pH = 4, pollutants concentration = 500 mg L^−1^).

The effect of adsorbent dose on the removal of phenol was studied by varying the dose of adsorbent from 0.05 to 0.35 g L^−1^ (Fig. 6). The adsorption capacity of phenol removal steeply increased with the adsorbent loading up to 0.35 g L^−1^. The increased adsorption of phenol by increasing the amount of adsorbent is due to an increase in the active sites and effective surface in the adsorbent.

### Adsorption isotherms

The adsorption isotherm is principally described as how solutes interact with adsorbents and optimize the critical adsorption capacity. Therefore, adsorption data of phenol on CMCM-48 were employed to test Langmuir, Freundlich, and Dubinin–Radushkevich isotherm models, used to explain the relationship between the amount (*q*_e_) adsorbed by the phenol and the concentration of the equilibrium (*C*_e_).

### Langmuir isotherm

The Langmuir isotherm assumed that the physical monolayer adsorption takes place at a finite number of homogeneous sites with the adsorbent.^24^ Then it is assumed that when phenol occupies a site, no further adsorption can take place at the surface.

It is generally applicable for homogenous surface. The general form of the expression is given by eqn (3)3
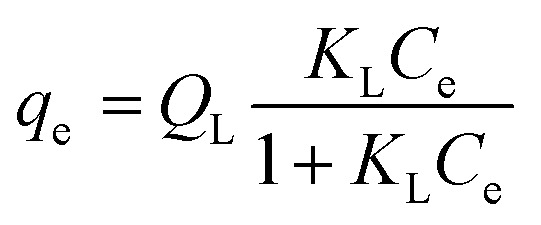
where, *C*_e_ was the equilibrium concentration (mg L^−1^), *Q*_L_ (mg g^−1^) and *K*_L_ (L mg^−1^) are the Langmuir's constants.

The linear Langmuir adsorption form can be represented as follows:4
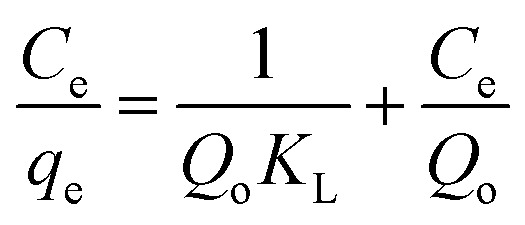
where *C*_e_ (mg L^−1^) is the equilibrium concentration of the adsorbate, *q*_e_ (mg g^−1^) is the phenol adsorbed amount per unit mass of adsorbent, *Q*_o_ is associated with adsorption capacity and *K*_L_ is Langmuir constant which determines the energy of adsorption (L mg^−1^).

**Fig. 7:**
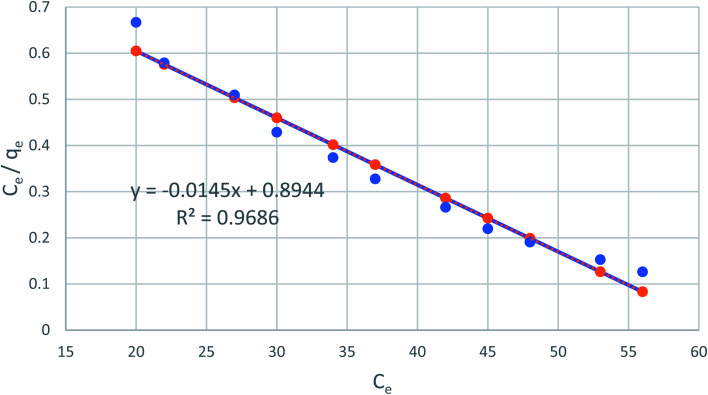
The Langmuir adsorption isotherm of phenol on CMCM-48 at 25.5 °C.

Fig. 7 shows the linear relationship of *C*_e_/*q*_e_*versus C*_e_ using the obtained experimental data, which suggests the Langmuir model applicability (*R*^2^ = 0.98), which is monolayer adsorption at the outer surface of the adsorbent. The constants of *Q*_o_ and Calculated from the slope and intercept of the plot, respectively are listed in Table 1.

**Table:** Data showing the values of the linear adsorption isotherms obtained for phenol adsorption on CMCM-48 nanocomposite

Isotherm equation	CMCM-48
Temperature	298

**Langmuir**	
*Q*_o_ (mg g^−1^)	69.0151
*K*_L_ (L mg^−1^)	0.0162
*R* ^2^	0.968635
*R*_L_	0.11–0.55

**Freundlich**	
K_f_ (mg g^−1^)	0.012474
*n*	0.391051
*R* ^2^	0.984616

**D–R**	
*q*_m_ (mg g^−1^)	365.7985
*β* (mol^2^ kJ^−2^)	0.000196
*E* (kJ mol^−1^)	−50.4713
*R* ^2^	0.85714

The essential characteristics of the Langmuir isotherm can be defined in terms of a dimensionless separation factor (*R*_L_) identified as:5
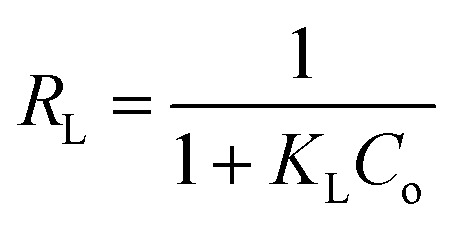
where *K*_L_ is the Langmuir constant and (*C*_o_) is the maximum phenol concentration (mg L^−1^). The value of *R*_L_ illustrates the type of the isotherm to be either favorable (0 < *R*_L_ < 1), unfavorable (*R*_L_ > 1), linear (*R*_L_ = 1), or irreversible (*R*_L_ = 0). The *R*_L_ value was estimated to be 0.0309 and this again confirmed that the Langmuir adsorption isotherm was favorable for phenol adsorption onto CMCM-48 under the used conditions in this study.

### Freundlich isotherm

Freundlich isotherm is commonly described as the multilayer adsorption of phenol occurs on a heterogeneous surface and the phenol adsorbed amount increases with increasing concentration.^25^

The Freundlich isotherm fit an empirical equation expressed as:

6 *q*_e_ = *K*_F_*C*^1/*n*^_e_

where *K*_F_ is roughly an indicator of the adsorption capacity for phenol and (1/*n*) of the adsorption intensity. The value of *n* > 1 represents a favorable adsorption condition. The linear Freundlich form of eqn (6) is:7
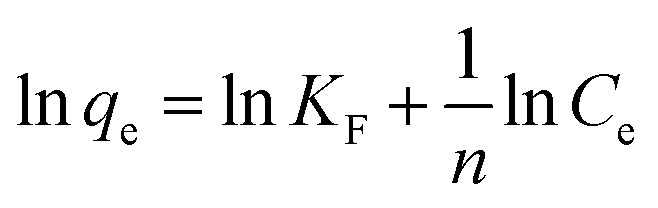


**Fig. 8:**
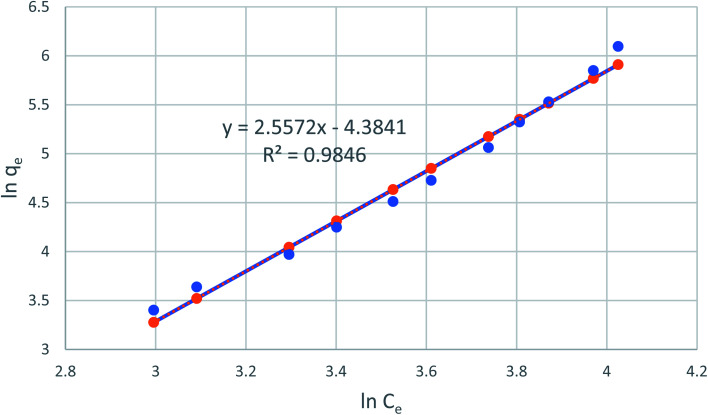
Freundlich adsorption isotherm of phenol onto CMCM-48 at 25.5 °C.

Fig. 8 reveals the straight line with a slope of (1/*n*) and an intercept of ln(*K*_F_) when ln(*q*_e_) is plotted *versus* ln(*C*_e_). The value of *n* was estimated to be 2.48, indicating that the favorable physical adsorption. Vander-Waal's forces involved in adsorption.

### Dubinin–Radushkevich isotherm model

**Fig. 9:**
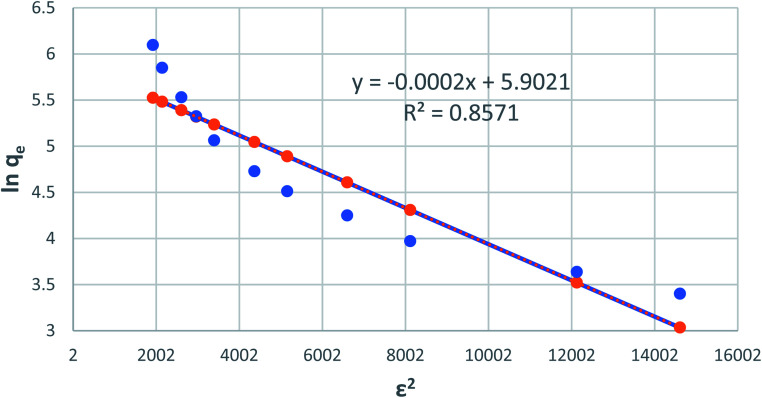
Dubinin–Radushkevich adsorption isotherm of phenol onto CMCM-48 at 25.5 °C.

Dubinin–Radushkevich isotherm is generally expressed as the Gaussian energy distribution with adsorption mechanism onto a heterogeneous surface.^24,26^ The model has successfully fitted the intermediate-range and maximum solute activities of concentrations data well. The linear D–R isotherm form is given as:

8 ln *Q*_e_ = ln *Q*_m_ − *βε*^2^

where *q*_e_ is the phenol adsorbed amount per unit mass of adsorbent (mg g^−1^), *Q*_m_ is theoretical isotherm saturation capacity (mg g^−1^), *β* is the constant of the sorption energy (mol^2^ kJ^−2^), and *ε* is Polanyi potential, which is described as9
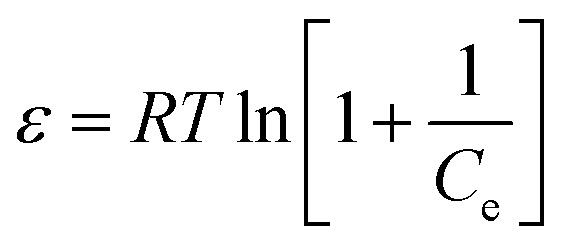

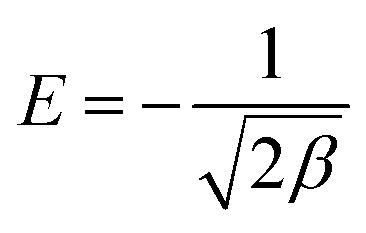
where *T* is the temperature of the solution (K) and *R* is the gas constant and is equal to 8.314 J mol^−1^ K^−1^. The values of *q*_m_ and *β* are calculated from the plot, as shown in Fig. 8 plotting of ln *q*_e_*versus ε*^2^, are illustrated in Table 1 and Fig. 9.

**Fig. 10:**
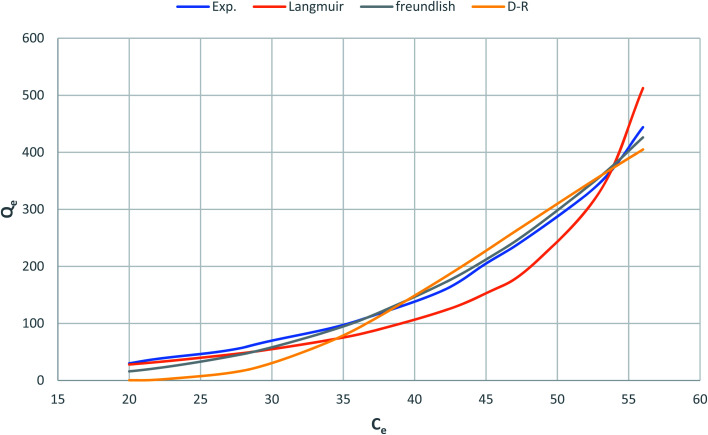
Nonlinear plot of adsorption isotherm models for phenol adsorption onto CMCM-48 nanocomposite.

Fig. 10 shows Langmuir, Freundlich and Dubinin–Radushkevich adsorption isotherms of the phenol by nonlinear analysis while Table 2 shows the values of the corresponding isotherm parameters and the correlation coefficients (*R*^2^) for each parameter. In Table 2 and Fig. 10, high *R*^2^ value are obtained by fitting the experimental data into the Langmuir isotherm model (*R*^2^ > 0.973) and the Dubinin–Radushkevich isotherm model (*V* > 0.946), as compared with the Freundlich isotherm model (*R*^2^ > 0.858). These suggest that both Langmuir and Dubinin–Radushkevich isotherm models can satisfactory fit the experimental data, while Freundlich isotherm model cannot. As shown, the values of maximum adsorption capacity determined using the Langmuir model was 59.5 mg P g^−1^ for CMCM-48. These values are near the experimental adsorbed amounts and correspond closely to the adsorption isotherm plateau, which indicates that the modeling of Langmuir for the adsorption system is acceptable. Moreover, the adsorption mechanism of the experimental system might be caused by the monolayer adsorption. The values of the theoretical monolayer saturation capacity in the Dubinin–Radushkevich model, obtained using non-linear regression, are all lower than the experimental amounts corresponding to the adsorption isotherm plateau, indicating that the modeling of Dubinin–Radushkevich for the adsorption system is unacceptable. Therefore, by comparison, the order of the isotherm best fits the four sets of experimental data in this study is Langmuir > Dubinin–Radushkevich > Freundlich.

Data showing the values of the non-linear adsorption isotherms obtained for phenol adsorption on CMCM-48 nanocomposite

**Table:** 

Isthermal model				
Langmuir model	*Q*_max_	*K*_L_	*R* ^2^	*R*_L_
	59.5	0.016	0.973328	1.0

**Table:** 

Isthermal model			
Freundlich isotherm	*K*_F_	*n*	*R* ^2^
	0.001132	0.313542	0.858497

**Table:** 

Isthermal model				
D–R model	*q*_m_	*β*	*R* ^2^	*E*
	1171.557	0.000552	0.945618	30.0861

### Adsorption kinetics

Kinetic studies predict adsorption rate constants such as adsorption mechanism and adsorption efficiency. The capability of the pseudo-first and pseudo-second-order kinetic models was evaluated at a constant temperature of 25.5 °C.

The kinetic adsorption data were treated with pseudo-first-order kinetic model:10
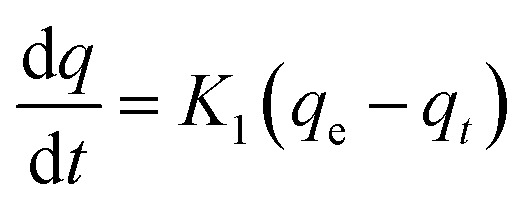
where *q*_e_ and qt refer to the adsorption capacity (mg g^−1^) at equilibrium and at any time, *t* (h), respectively, and *k*_1_ is the pseudo-first-order sorption rate constant of (h^−1^). Integration of eqn (10) for the boundary conditions *t* = 0 to *t* and *q*_*t*_ = 0 to *q*_t_, gives eqn (11) below:11
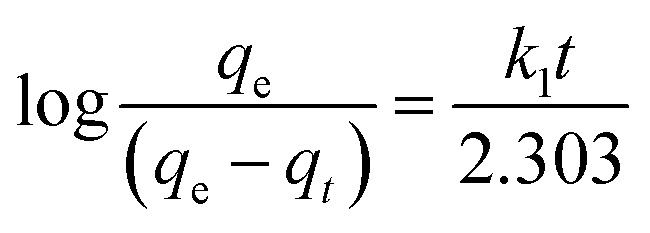


Eqn (11) can be rearranged to give eqn (12):12
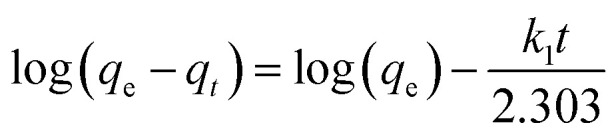


**Fig. 11:**
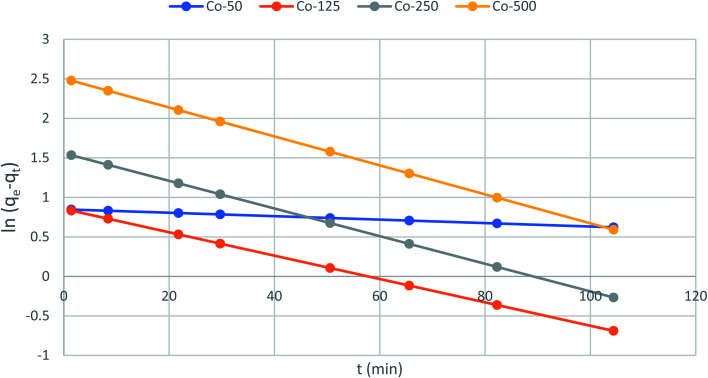
Pseudo-first-order kinetics for adsorption of phenol onto CMCM-48 (initial concentration = 500 mg L^−1^, the adsorbent dosage = 0.1 g, temperature = 25.5 °C).

The slope and intercept of the graph of log (*q*_e_ − *q*_*t*_) *versus t* (Fig. 11) were used to estimate the first-order rate constant *k*_1_. It can be seen from the results that the experimental (*q*_e_) values do not match well with the measured ones, which means that the application of the pseudo-first-order isotherm to the adsorption of the phenol cycle is not feasible.

The kinetic data were further analyzed with the pseudo-second-order kinetics. The pseudo-second-order equation is also based on the sorption capacity rate, it provides the best correlation of the experimental data and the adsorption's mechanism is chemically rate controlling.

The equation of pseudo-second-order kinetics is expressed as follows:^27^13
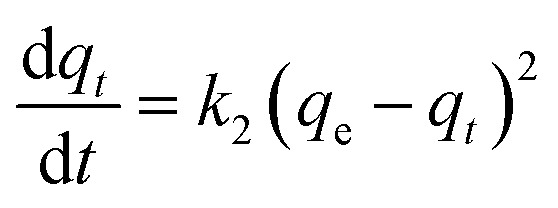
where *k*_2_ is the pseudo-second-order adsorption rate constant (g mg^−1^ h^−1^). Integrating eqn (13) for the boundary condition *t* = 0 to *t* and *q*_*t*_ = 0 to *q*_*t*_, gives eqn (14):14
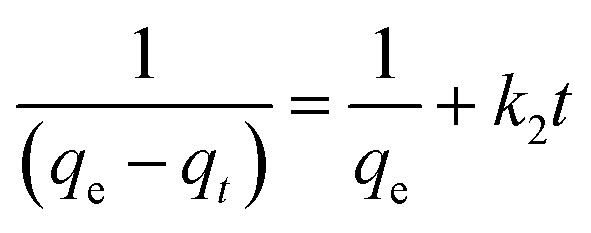


Which is the integrated rate law for a pseudo-second-order reaction. Eqn (14) can be rearranged to obtain a linear form as eqn (15):15
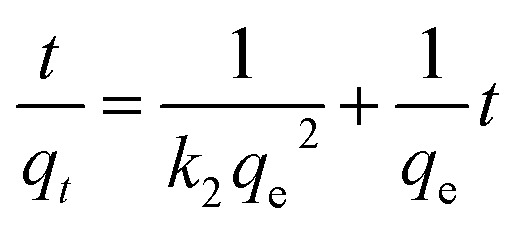


The second-order rate constant k_2_can be obtained from the slope and intercept of the plot of *t*/*q*_*t*_*versus t* (Fig. 11). The nonlinear equations are expressed in Table 3. The results of the rate constant studies for different initial phenol concentrations by the pseudo-first-order and second-order models and their linear and nonlinear forms are listed in Table 4.

**Table:** The nonlinear equations for Pseudo-first and second-order kinetics

Isotherm	Nonlinear form
Pseudo first-order	*q*_*t*_ = *q*_e_(1−e^−*k*_1_^)
Type 2, pseudo second-order	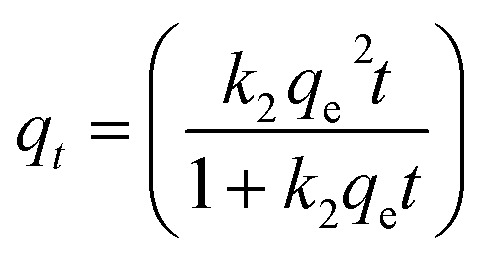

**Table:** Kinetics parameters for the adsorption phenol on CMCM-48 nanocomposite

*C*_o_ (mg L^−1^)	*q*_e_,_exp_ (mg g^−1^)	Pseudo-first-order kinetic model			Pseudo-second-order kinetic model		
		*q*_e_,_cal_ (mg g^−1^)	*k*_1_ (h^−1^)	*R* ^2^	*q*_e_,_cal_ (mg g^−1^)	*k*_2_ (g mg^−1^ h^−1^)	*R* ^2^
**Linear adsorption kinetics models**							
50	42	49	0.654	0.587	48	0.258	0.999
100	78	20	0.321	0.633	97	0.078	0.999
250	200	100	0.957	0.959	248	0.024	0.999
500	300	120	0.456	0.966	422	0.047	1.000

**Nonlinear adsorption kinetics models**							
50	42	50	0.214	0.687	50.2	0.654	0.995
100	78	22	0.376	0.423	99	0.341	0.989
250	200	111	0.321	0.979	255	0.432	0.969
500	300	132	0.567	0.826	472	0.875	0.999

**Fig. 12:**
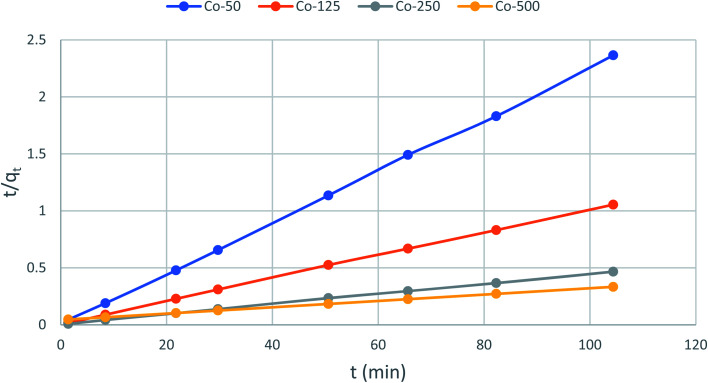
Pseudo-second-order kinetics for adsorption of phenol onto CMCM-48 (initial concentration = 500 mg L^−1^, the adsorbent dosage = 0.1 g, temperature = 25.5 °C).

From the analysis, it can be seen that the *q*_e_ (experimented) values are relatively similar to those of the *q*_e_ (calculated) values for the pseudo-second order. It can therefore be inferred that the adsorption of phenol to CMCM-48 nanocomposite is represented in a pseudo-second-order kinetic model. As well as the concern of *R*^2^, the applicability of the kinetic models was further proved through normalized standard deviation Δ*q* (%) defined as: 16
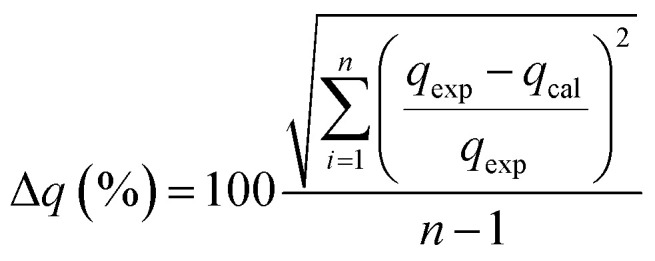
where the subscripts ‘cal’ and ‘exp’ refer to the calculated and experimental values, respectively and *n* is the number of data points. The higher the *R*^2^ value and the lower the Δ*q* (%) value the better a match is indicated. Table 2 lists the values acquired for the Δ*q* (%). The results recommended that the overall rate of the phenol adsorption process appeared to be controlled by the chemisorption process and the pseudo-second-order adsorption mechanism was predominant (Fig. 12).

### Intraparticle diffusion modeling

**Fig. 13:**
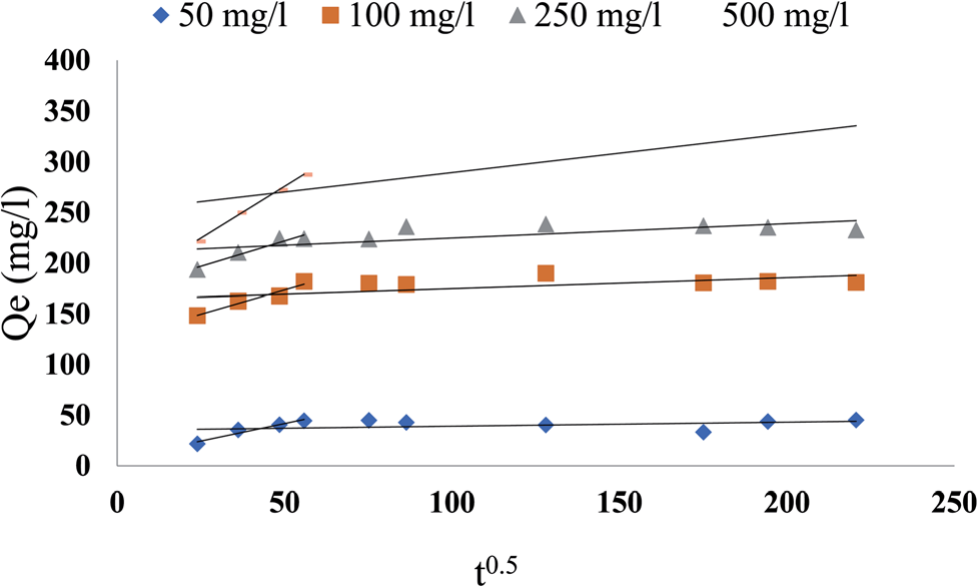
Intra-particle diffusion plot for phenol adsorption at 25.5 °C on CMCM-48 for various initial concentrations of phenol.

Based on the above findings, neither the pseudo-first-order nor the second-order model can evaluate the diffusion mechanism, the kinetic effects have been defined by the intraparticle diffusion model to reveal the diffusion mechanism, which is represented as:

17 *q* = *k*_i_*t*^0.5^ + *C*

twhere *q* is the phenol adsorbed amount (mg g^−1^) at time *t*, *k*_i_ is intraparticle diffusion constant (mg g^−1^ min^−0.5^), and *c* is the intercept. The constants were calculated and listed in Table 5. If the *c* value is zero, the adsorption rate is organized by the intraparticle diffusion for the entire adsorption period. Though, the plot of *q* against *t*^0.5^ usually shows more than one linear component, and if the slope of the first component is not zero, the diffusion of the boundary layer at the beginning controls the rate of adsorption. As shown in Fig. 13, the plots were not linear over an all, which meant more than one process had an adsorption effect. The related phenol adsorption pattern on CMCM-48 at different initial concentrations is among the key observations. The multiple nature observed in the intraparticle diffusion plot has been reported to indicate that intraparticle diffusion is not strictly rate-controlling. In addition, external mass transfer of phenol molecules to sorbent particles, particularly during the initial reaction phase, was also significant in the sorption process.

**Table:** Constant intraparticular diffusion for different initial concentrations of phenols

Linear model			Nonlinear model		
*C*_o_ (mg L^−1^)	*k*_i1_ (mg g^−1^ h^−0.5^)	*R* ^2^	*C*_o_ (mg L^−1^)	*k*_i1_ (mg g^−1^ h^−0.5^)	*R* ^2^
50	12.4	0.958	50	12.9	0.932
100	41.02	0.988	100	43.11	0.954
250	16.4	0.956	250	17.01	0.987
500	19.2	0.928	500	20.11	0.988

**Fig. 14:**
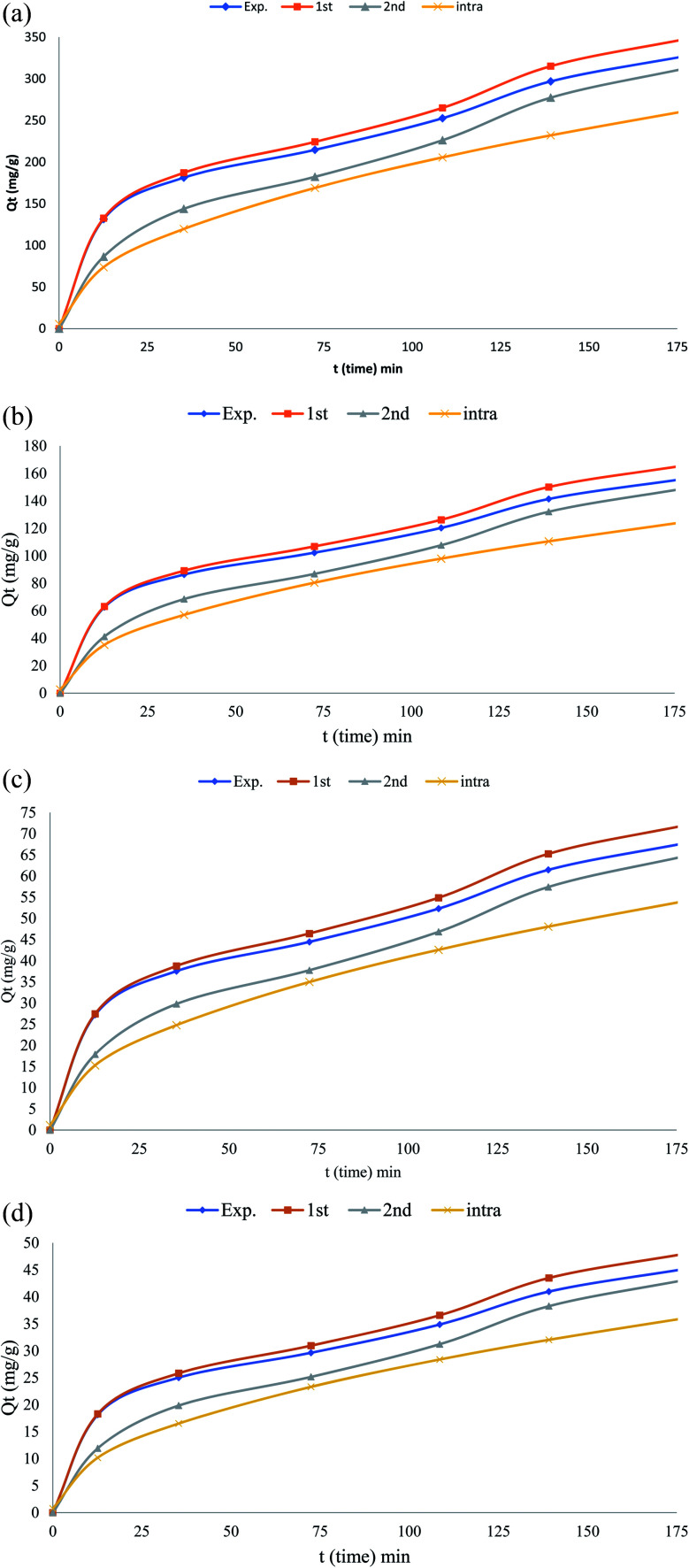
(a) Nonlinear adsorption kinetics of phenol on the CMCM-48 nanocomposite (initial concentration = 50 mg L^−1^, the adsorbent dosage = 0.1 g, temperature = 25.5 °C), (b) nonlinear adsorption kinetics of phenol on the CMCM-48 nanocomposite (initial concentration = 125 mg L^−1^, the adsorbent dosage = 0.1 g, temperature = 25.5 °C), (c) nonlinear adsorption kinetics of phenol on the CMCM-48 nanocomposite (initial concentration = 250 mg L^−1^, the adsorbent dosage = 0.1 g, temperature = 25.5 °C), (d) nonlinear adsorption kinetics of phenol on the CMCM-48 nanocomposite (initial concentration = 500 mg L^−1^, the adsorbent dosage = 0.1 g, temperature = 25.5 °C).

The kinetic isotherm parameters were obtained using the linear least-squares regression method and trial-and-error nonlinear regression method in the Microsoft Excel. The nonlinear and the linearized expressions of the pseudo-first^22^ and pseudo-second order kinetic^22^ isotherms are shown in Tables 2 and 4 The lower *R*^2^ value shows that pseudo-first order kinetic model was not an appropriate to explain the sorption kinetics. The pseudo-second order kinetic model could be linearized and simple linear regression would result in different parameter estimates.^28^ In nonlinear method, “solver add-in” of Microsoft Excel was used for determining the pseudo-first order and pseudo second-order kinetic parameters by trial-and-error. Experimental data with the pseudo-first order and pseudo second kinetic model obtained by using the nonlinear method for the sorption of phenol using CMCM-48 was shown (Fig. 14 a–d). It was observed that the nonlinear pseudo first order equation exhibited higher *R*^2^ values than the linear form (Table 4). Moreover, the calculated *q*_e_ value from non-linear pseudo-first order equation was very close to the experimental *q*_e_ value. The best and worse fit of experimental kinetic data in pseudo first-order kinetics by both nonlinear and linear methods suggested that kinetics was transforming to the worse while linearizing the non-linear pseudo first-order kinetics expression. Hence linear pseudo-first order kinetic expression would be inappropriate to check whether the experimental kinetic was following a first-order kinetics and leaving the nonlinear first-order expression as a better option. From Table 4, it was observed that the kinetic parameters associated with the pseudo-second-order model calculated by linear and nonlinear methods varied a little. No problem was found while using nonlinear method regarding transformations of nonlinear pseudo-second order equation to linear forms, and also they were in the same error structures. Though the values of *R*^2^ for nonlinear pseudo second order was almost similar to nonlinear pseudo-second order, but the calculated *q*_e_ from nonlinear pseudo-second order was more close to the experimental *q*_e_ value than the difference between *q*_e,exp_ to *q*_e,cal_ of linear expression. This simply showed that nonlinear model had an advantage over the linear model (Table 4).

### Reusability of adsorbent

**Fig. 15:**
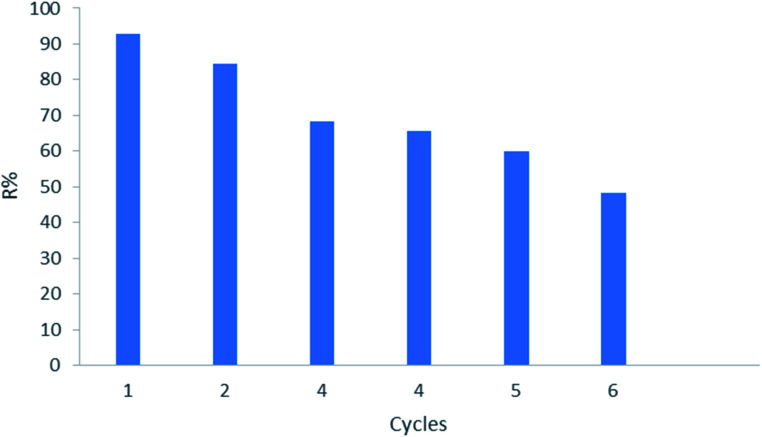
Reuse effect ofCMCM-48 for the adsorption of phenol (initial concentration = 500 mg L^−1^, contact time = 120 min, the adsorbent dosage = 0.1 g, temperature = 25.5 °C).

To determine the likelihood of CMCM-48 adsorbent being regenerated and reused, six cycles of adsorption–desorption were performed using a phenol solution. Desorption behavior was performed under optimum conditions using an aqueous solution of (anhydrous ethanol : water = 2 : 1). Fig. 15 shows that in six cycles, the removal rate of phenol (*R*%) gradually decreased from 92.94% to 48.13% for phenol solution. The large decrease of removal due to the loss of the associated adsorbent material during the repeated cycles. These results indicate that CMCM-48 adsorbent could perform effectively for reusability even after six cycles.

### Comparison with other studies

Table 6 reveals that the adsorption efficiency of CMCM-48 is higher than the majority of adsorbents. In short, taking into account the process characteristics of this work, CMCM-48 as a low-cost sorbent not only decreases the amount of chitosan required for cheap use, but also has a high adsorption capacity and good mechanical stability, which recognizes that CMCM-48 is a decent choice for economically viable.

**Table:** Maximum adsorption capacity (*q*_max_) values of phenol on CMCM-48 compared with other reported adsorbents in the literature

Adsorbent	*q*_max_ (mg g^−1^)	Ref.
WPT-CTAB	0.05	29
Sawdust	2	30
Eggshell waste	7.63	31
MMT–CTAB	10.48	29
magnetic *N*-methyl-d glucamine	13.44	32
Natural clay	15	33
Diethylenetriamine–ctivated carbon	18.12	34
Magnetic biosorbent	59	
Ca–bentonite–chitosan	125	35
β-Cyclodextrin–chitosan	92.18	36
GA–chitosan	51.68	37
MCM-48–chitosan	149.25	This work

## Conclusion

The removing of phenol from aqueous solutions using chitosan-based MCM-48 nanocomposite as adsorbent was investigated using batch processing. The results illustrate the effect of the experimental parameters on the ability of adsorption for phenol, such as adsorbent dosage, pH value, initial concentrations, and contact time. The maximum adsorption capacity to remove phenol of 149.25 mg g^−1^ occurred at pH = 8. Equilibrium studies indicate that the data fit very well in the Dubinin–Radushkevich model based on the correlation coefficient (*R*^2^ = 0.983085). The adsorption kinetics indicated that the pseudo-second-order isotherm model is better described in the adsorption process. The desorption studies conducted using phenol demonstrated the positive potential of CMCM-48 adsorbent regeneration and reuse, the adsorbents used in this study give many promising advantages for commercial purposes in the future. The results verified that the CMCM-48 prepared is economically promising for wastewater treatment.

## Conflicts of interest

There are no conflicts to declare.

## Supplementary Material
